# Adiposity and domain-specific menopausal symptom severity in midlife women: a cross-sectional clinical study in eastern china

**DOI:** 10.3389/fmed.2026.1831154

**Published:** 2026-05-22

**Authors:** Miao Deng, Hongyan Zhang, Zhifen Zhang, Nan Chen

**Affiliations:** Reproductive Endocrine Center, Hangzhou Matenal and Child Health Care Hospital (Hangzhou Womens Hospital), Hangzhou, Zhejiang, China

**Keywords:** body mass index, cross-sectional study, menopause, midlife women, obesity, symptom severity, women’s health

## Abstract

**Objective:**

To investigate the association between body mass index (BMI) and the prevalence and severity of menopausal symptoms in a clinically characterized cohort of peri- and postmenopausal women in Eastern China.

**Methods:**

In this cross-sectional study, 1,371 women aged 40–60 years were recruited from the Perimenopausal Health Care Center of Hangzhou Women’s Hospital, Zhejiang University School of Medicine, between June 2022 and June 2025. Anthropometric measurements were obtained using standardized protocols, and BMI categories were defined according to Chinese guidelines. Menopausal symptoms were evaluated using the modified Kupperman Menopausal Index. Differences across BMI groups were assessed using analysis of variance or chi-square tests, and correlations between BMI and symptom severity were examined using Pearson correlation analysis.

**Results:**

Vasomotor symptoms were the most prevalent menopausal complaints in this cohort, reported by 76.6% of participants. High frequencies were also observed for sexual dysfunction (72.4%), fatigue (72.0%), insomnia (71.8%), and mood swings (66.3%). Although the overall prevalence of menopausal symptoms did not differ significantly across BMI categories, symptom severity was greater in several domains. Specifically, there were significant positive correlations between BMI and vasomotor symptoms, mood swings, sexual dysfunction, and urinary symptoms. These associations remained statistically significant but modest in multivariable analyses adjusting for waist-to-hip ratio. Consistently, women with obesity had significantly higher scores for vasomotor, sexual, and urinary symptoms compared with women of normal weight.

**Conclusions:**

While BMI was not associated with overall symptom prevalence, it was associated with modest increases in the severity of selected symptom domains. These findings should be interpreted cautiously given the small effect sizes and cross-sectional design. Clinical trial number: not applicable.

## 1 Introduction

Menopause represents a major biological transition that affects the health and quality of life of millions of women worldwide (1). In East Asia, approximately 60–80% of women report moderate-to-severe menopausal symptoms, with vasomotor symptoms, sexual dysfunction, and urinary complaints among the leading contributors to reduced health-related quality of life (2, 3). These symptoms frequently coexist with increased risks of cardiometabolic disease, osteoporosis, and neurocognitive decline. Symptom expression during the menopausal transition is shaped by a complex interplay of genetic, endocrine, metabolic, and psychosocial factors, and emerging evidence suggests that adiposity may represent a potentially modifiable determinant of symptom burden (4).

The menopausal transition is characterized by progressive ovarian senescence and declining estrogen levels, leading to multiple systemic physiological changes (5). These hormonal alterations influence thermoregulatory control, vascular reactivity, and urogenital tissue integrity, resulting in vasomotor symptoms, genitourinary syndrome of menopause, sexual dysfunction, and mood or cognitive disturbances (6). Excess adiposity, commonly assessed using body mass index (BMI) and central fat distribution indices such as waist-to-hip ratio (WHR), may exacerbate these symptoms (6–8).

Several biological mechanisms may explain the relationship between adiposity and the severity of menopausal symptoms. Adipose tissue expresses aromatase, which increases peripheral estrogen production, while obesity-related inflammatory cytokines such as tumor necrosis factor-α and interleukin-6 may disrupt hypothalamic–pituitary–ovarian axis signaling (9, 10). In addition, obesity is associated with endothelial dysfunction, impaired nitric oxide–mediated vasodilation, and autonomic imbalance, all of which may influence thermoregulatory and urogenital function. Collectively, these mechanisms may relate to the severity of vasomotor and genitourinary symptoms during the menopausal transition (11).

Epidemiological studies investigating the association between BMI and menopausal symptoms have produced inconsistent findings. While several studies conducted in Western populations report a positive association between higher BMI and vasomotor symptom severity, some studies in Asian populations have observed weaker or even inverse relationships (12, 13). These discrepancies may reflect differences in genetic background, fat distribution patterns, dietary phytoestrogen intake, environmental factors, and cultural variations in symptom perception or reporting. Differences in methodology, including inconsistent staging, variable symptom measures, and limited confounder adjustment, may explain this uncertainty (12).

Despite increasing interest in the relationship between adiposity and menopausal symptoms, relatively few studies have simultaneously examined multiple symptom domains, including vasomotor, sexual, and urinary symptoms, within clinically characterized menopausal populations in East Asia (14). In China, menopause research has expanded alongside demographic aging; however, much of the existing evidence is derived from community-based surveys that lack standardized clinical phenotyping.

To address these gaps, we analyzed a large, clinically verified cohort of women undergoing the menopausal transition in Eastern China (2, 12). We hypothesized that BMI would be related to greater severity in specific menopausal domains, especially vasomotor and sexual symptoms. By examining a clinically characterized population, this study aims to clarify the relationship between adiposity and menopausal symptom burden and to provide evidence that may inform preventive and lifestyle-based strategies to improve health outcomes among midlife women.

## 2 Material and methods

### 2.1 Study population

This cross-sectional study was conducted at the Perimenopausal Health Care Center of Hangzhou Women’s Hospital, between June 2022 and June 2025. A total of 1,468 women seeking evaluation for perimenopausal or menopausal health concerns were screened. Ninety-seven women were excluded due to incomplete baseline demographic information or missing Modified Kupperman Index (mKI) scores, leaving 1,371 participants for the final analysis.

Eligible participants were women aged 40–60 years (mean ± SD, 49.89 ± 5.18 years) who had resided in Hangzhou for at least 10 years. They met the Stages of Reproductive Aging Workshop + 10 (STRAW + 10) criteria for menopausal transition or natural menopause, defined by menstrual cycle changes or ≥ 12 months of amenorrhea, with or without menopausal symptoms (15). All participants provided written informed consent and completed clinical evaluation, anthropometric measurements, and symptom assessment during a single study visit. Women were excluded if they had major systemic diseases (including uncontrolled cardiovascular disease, chronic renal or hepatic insufficiency, or active malignancy), endocrine disorders affecting menopausal status (such as pituitary adenoma, hyperprolactinemia, or untreated thyroid disease), diagnosed psychiatric illness requiring medication, significant life stressors within the preceding 6 months, current or recent (within 6 months) use of menopausal hormone therapy, selective estrogen receptor modulators, or phytoestrogen supplements, a history of hysterectomy or bilateral oophorectomy, or incomplete or unreliable questionnaire data (14). These criteria were applied to ensure a clinically homogeneous cohort and to minimize potential confounding factors related to comorbidities or recent therapeutic interventions (16).

### 2.2 Data collection

All participants underwent structured, interviewer-administered assessments conducted by physicians trained in standardized interviewing procedures. The questionnaire was adapted from a validated instrument described in our previous publication (14). Menopausal symptoms were assessed using the mKI, which has been described in detail in the Chinese version of the Menopause Rating Scale (MRS) and related validation studies (14, 17, 18). In addition to demographic and menstrual history, menopausal symptom severity was assessed using the Chinese versions of the MRS and the mKI (14, 16), a validated composite scale comprising 13 items grouped in to four primary domains: vasomotor complaints (hot flashes, sweating), mood swings (including depressive mood, irritability, and insomnia), sexual dysfunction, and urinary symptoms. Additional somatic and neurological complaints included paranesthesia, dizziness, fatigue, arthralgia or myalgia, headache, palpitations, and formication. Each item was assigned a weight score (1–4) and rated for severity (0 = none to 3 = severe). Domain and total mKI scores were calculated as the product of weight and severity for each symptom, summed across relevant items (19). The total mKI score represents the sum of all individual weighted symptom scores, with higher scores indicating greater overall symptom severity.

Anthropometric measurements, including height, weight, waist circumference, and hip circumference, were obtained in the morning after an overnight fast, with participants wearing light indoor clothing and no shoes. Height and weight were measured using a calibrated stadiometer and digital scale, and BMI was calculated as weight in kilograms divided by height in meters squared. Waist circumference was measured at the midpoint between the lower margin of the last palpable rib and the iliac crest, while hip circumference was measured at the level of the greatest gluteal protrusion in alignment with the pubic symphysis. WHR was calculated as waist circumference in centimeters divided by hip circumference in centimeters. BMI categories were defined by the Chinese Guidelines for the Prevention and Control of Overweight and Obesity in Adults (2024 edition). Underweight was defined as BMI < 18.5 kg/m^2^, normal weight as 18.5–23.9 kg/m^2^, overweight as 24.0–27.9 kg/m^2^, and obesity as ≥ 28.0 kg/m^2^. These standardized measurement protocols ensured consistency across participants and allowed for reliable stratification of adiposity status regarding menopausal symptom burden.

### 2.3 Statistical analysis

Data analysis was carried out using SPSS software (version 22.0; IBM Corp., Armonk, NY, United States). Before analysis, the distribution of continuous variables was examined using the Shapiro–Wilk test together with visual inspection of histograms. Equality of variances was assessed using Levene’s test. Variables with an approximately normal distribution are reported as mean ± standard deviation, while skewed variables are presented as median with interquartile range. Categorical variables are expressed as numbers and percentages. Comparisons of continuous variables among BMI groups were performed using one-way analysis of variance when distributional assumptions were met. In cases where normality or variance assumptions were not satisfied, the Kruskal–Wallis test was used instead. *Post hoc* comparisons were conducted as appropriate. Differences in categorical variables were assessed using the chi-square test. The relationships between anthropometric measures (BMI and WHR) and menopausal symptom burden, including total mKI scores and individual symptom components, were evaluated using Pearson correlation for normally distributed variables and Spearman rank correlation for non-normally distributed variables. Parametric tests were applied based on approximate normal distribution of the data, as assessed by normality testing. A two-sided *p*-value of less than 0.05 was considered statistically significant. Figures were prepared using GraphPad Prism (version 5.0; GraphPad Software, La Jolla, CA, United States). Multivariable linear regression analyses were performed to assess the independent association between BMI (continuous) and domain-specific menopausal symptom scores, adjusting for WHR. Separate models were constructed for each symptom domain, and regression coefficients (β), 95% confidence intervals (CIs), and corresponding *p*-values were reported.

## 3 Results

### 3.1 General characteristics and anthropometric findings

The study included 1,371 perimenopausal women classified according to WHO Asian BMI criteria as underweight (*n* = 63), normal weight (*n* = 794), overweight (*n* = 308), and obese (*n* = 206). Mean age differed significantly across BMI categories (*F* = 17.334, *P* < 0.001), increasing from 48.10 ± 4.39 years in the underweight group to 51.38 ± 2.90 years in the obese group. WHR showed a similar pattern (*F* = 57.548, *P* < 0.001), rising progressively from 0.81 ± 0.05 in underweight women to 0.88 ± 0.05 in obese women (all pairwise *P* < 0.001), indicating increasing central adiposity across BMI categories.

Prevalence data presented in Supplementary Table 1 further illustrate these anthropometric patterns. Vasomotor symptoms were highly prevalent across all BMI groups, affecting more than 70% of participants. Differences in prevalence were not statistically significant (*P* = 0.140); however, symptom severity tended to increase with BMI. A similar pattern was observed for sexual dysfunction, with prevalence numerically higher in obese women (78.64%) than in underweight women (66.67%), although this difference did not reach statistical significance (χ^2^ = 7.480, *P* = 0.059). Urinary symptoms showed a numerical increase across BMI groups, rising from 22.22% in the underweight group to 37.38% in the obese group (χ^2^ = 5.493, *P* = 0.139). Overall, these patterns paralleled the increases observed in BMI and WHR across the study groups. Overall, these patterns paralleled the increases observed in BMI and WHR across the study groups (Table 1).

**TABLE 1:** Anthropometric characteristics across BMI-defined study groups.

Variables	Underweight (*n* = 63)	Normal weight (*n* = 794)	Overweight (*n* = 308)	Obese (*n* = 206)
Age (yrs)	48.10 ± 4.39	50.06 ± 3.29*	49.98 ± 3.63*	51.38 ± 2.90**,^†,‡^
BMI (kg/m^2^)	17.64 ± 0.75	21.10 ± 1.13**	23.74 ± 0.52**,^††^	26.44 ± 1.36**,^††,‡‡^
WHR	0.81 ± 0.05	0.83 ± 0.05*	0.86 ± 0.05 *,^†^	0.88 ± 0.05**,^†,‡^

All data expressed as mean ± SD (range) of the mean of individual groups.

*P < 0.05 and **P < 0.001 vs. the underweight group.

† *P* < 0.05 and ^††^*P* < 0.001 vs. the normal group.

‡ *P* < 0.05 and ^‡‡^*P* < 0.001 vs. the overweight group. BMI groups were defined using WHO Asian cut-offs, enabling consistent comparison across body composition ranges. BMI, body mass index; WHR, waist-to-hip ratio.

### 3.2 Modified Kupperman index symptom findings

Domain-specific variations in mKI scores across BMI categories are shown in Table 2. Vasomotor symptom scores were highest in obese women (5.67 ± 3.93), significantly exceeding those observed in normal-weight and underweight participants (both *P* < 0.05). This was consistent with prevalence data showing that vasomotor complaints exceeded 79% among overweight and obese women. Sexual dysfunction showed a clear gradient across BMI groups, with mean scores increasing from 1.56 ± 1.27 in underweight women to 2.05 ± 1.55 in obese women (*P* < 0.05 for all comparisons). Prevalence data reflected a similar trend, increasing from 66.67% in underweight women to 78.64% in obese participants.

**TABLE 2:** Modified Kupperman Index symptom scores in perimenopausal women across BMI-defined groups.

Symptom	Underweight (*n* = 63)	Normal weight (*n* = 794)	Overweight (*n* = 308)	Obese (*n* = 206)
Vasomotor complaints	5.02 ± 3.66	4.95 ± 3.84	5.48 ± 3.79^†^	5.67 ± 3.93*^,†^
Paresthesia	0.92 ± 1.47	0.94 ± 1.48	0.90 ± 1.48	1.22 ± 1.62
Insomnia	21.10 ± 1.13**	2.13 ± 1.69	2.22 ± 1.72	1.90 ± 1.61
Mood swings	1.59 ± 1.25	1.63 ± 1.49	1.81 ± 1.47	1.87 ± 1.53
Depressive	0.56 ± 0.76	0.53 ± 0.72	0.61 ± 0.73	0.49 ± 0.69
Dizziness	0.63 ± 0.79	0.55 ± 0.66	0.56 ± 0.68	0.50 ± 0.68
Fatigue	1.03 ± 0.76	0.89 ± 0.68	0.88 ± 0.67	0.93 ± 0.69
Arthralgia	0.62 ± 0.71	0.84 ± 0.82	0.87 ± 0.83	0.77 ± 0.84
Headache	0.41 ± 0.61	0.46 ± 0.66	0.44 ± 0.64	0.48 ± 0.66
Palpitations	0.49 ± 0.67	0.52 ± 0.66	0.53 ± 0.67	0.55 ± 0.61
Formication	0.21 ± 0.48	0.18 ± 0.47	0.19 ± 0.45	0.17 ± 0.42
Sexual dysfunction	1.56 ± 1.27	1.76 ± 1.47	1.73 ± 1.29	2.05 ± 1.55*^,†,‡^
Urinary symptoms	0.67 ± 1.39	0.80 ± 1.31	0.86 ± 1.39	1.09 ± 1.64*^,†^

All data expressed as mean ± SD (range) of the mean of individual groups.

*P < 0.05 vs. the underweight group.

† *P* < 0.05 vs. the normal group.

‡ *P* < 0.05 vs. the overweight group. BMI groups were defined using WHO Asian cut-offs, enabling consistent comparison across body composition ranges; Sexual dysfunction: problems with sexual desire, arousal, or satisfaction; Urinary symptoms: urgency, frequency, or incontinence.

Urinary symptoms followed a comparable pattern, with mean scores increasing from 0.67 ± 1.39 in the underweight group to 1.09 ± 1.64 in the obese group (both *P* < 0.05). Prevalence increased from 22.22 to 37.38% across groups, without reaching statistical significance.

In contrast, other symptom domains, including paresthesia, dizziness, mood swings, fatigue, arthralgia, headache, palpitations, and formication, showed no significant differences in either severity or prevalence across BMI categories. Overall, these findings suggest that BMI is primarily associated with vasomotor symptoms, sexual dysfunction, and urinary symptoms, whereas other menopausal complaints appear largely independent of body composition.

### 3.3 Correlation findings

#### 3.3.1 Association between anthropometric characteristics and total menopausal symptom burden

**FIGURE 1:**
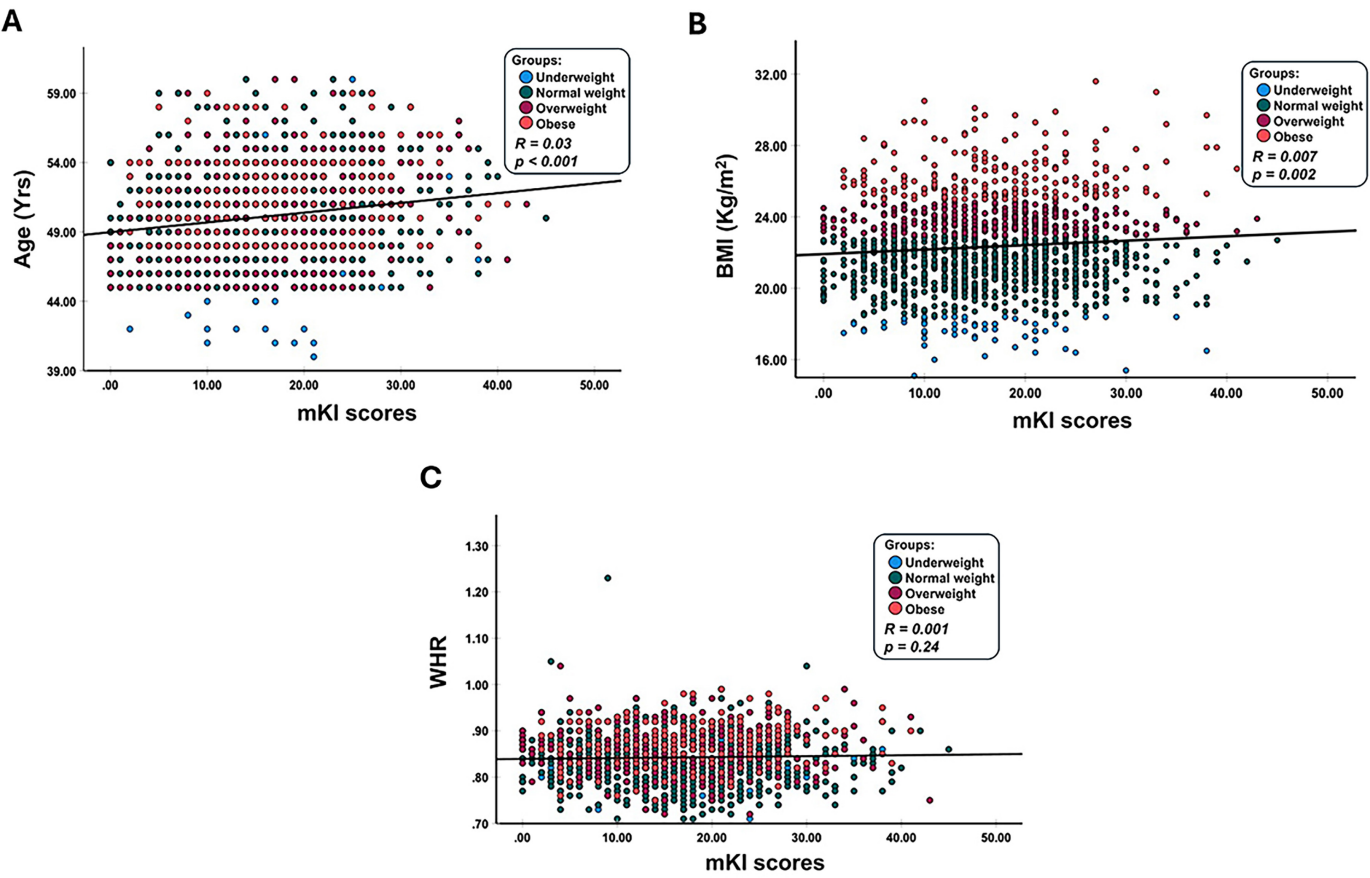
Correlation of chronological age, waist-to-hip ratio (WHR), and body mass index (BMI) with total menopausal symptom burden as assessed by the modified Kupperman Menopausal Index (mKI). Scatter plots depict the relationships between **(A)** chronological age, **(B)** WHR, and **(C)** BMI with the overall mKI score in a cohort of peri- and postmenopausal women (*n* = 1,371). Pearson’s correlation coefficients (r) and two-tailed *p*-values are indicated on each panel. Solid regression lines represent the best-fit linear model, and shaded areas indicate the 95% confidence interval for the regression estimate. The scatter of data points reflects variability in the observed relationships.

Correlation analyses were conducted to examine the relationships between chronological age, general adiposity, central adiposity, and overall menopausal symptom burden as measured by the mKI. In the overall cohort, chronological age demonstrated a statistically significant but weak positive correlation with total mKI score (*r* = 0.03, *P* < 0.001; Figure 1A). BMI showed a statistically significant but negligible correlation with total symptom burden (*r* = 0.007, *P* = 0.002; Figure 1B). In contrast, WHR was not significantly associated with total mKI score (*r* = 0.001, *P* = 0.24; Figure 1C). Adjusted associations were further evaluated using multivariable regression models. The wide dispersion of data points further reflects the weak strength of these associations. Taken together, these results indicate that age, BMI, and WHR were not meaningfully correlated with overall menopausal symptom burden in this cohort.

#### 3.3.2 Domain-specific correlations between BMI and menopausal symptom components

To further investigate potential associations between anthropometric characteristics and specific symptom domains, additional correlation analyses were conducted for each component of the mKI. Neither chronological age nor WHR demonstrated significant correlations with individual symptom domains.

**FIGURE 2:**
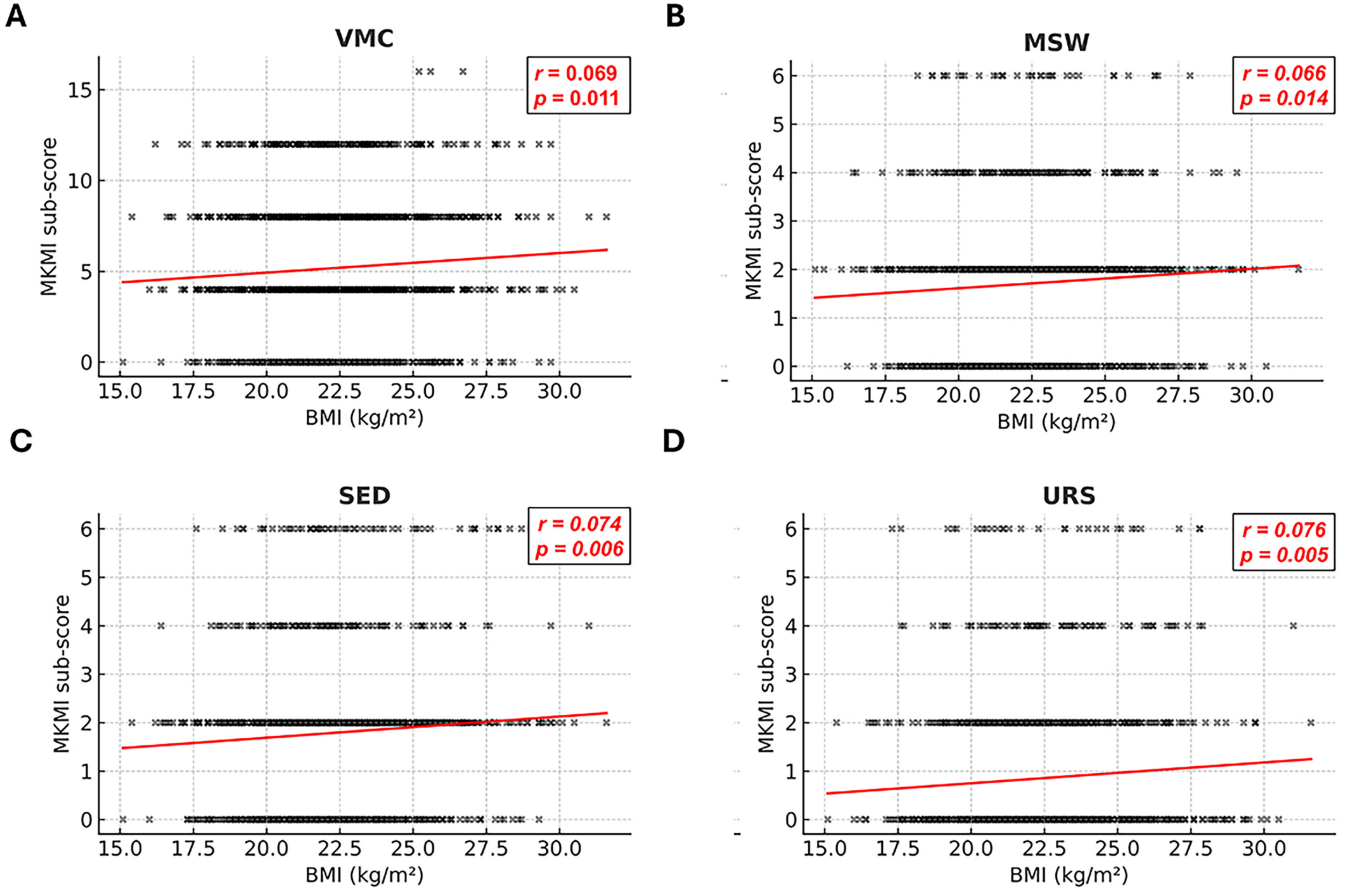
Domain-specific correlations between body mass index (BMI) and individual symptom domains of the modified Kupperman Menopausal Index (mKI). Panels show scatter plots illustrating Pearson’s correlations between BMI and **(A)** vasomotor complaints (VMC), **(B)** mood swings (MSW), **(C)** sexual dysfunction (SED), and **(D)** urinary symptoms (URS) in peri- and postmenopausal women (*n* = 1,371). Positive correlations were observed for all four domains: VMC, MSW, SED, and URS. Solid regression lines represent the best-fit linear relationship; shaded regions indicate the 95% confidence intervals. The distribution of data points indicates variability in the observed relationships.

In contrast, BMI showed statistically significant positive correlations with several symptom domains (Figure 2). Higher BMI was correlated with greater severity of vasomotor complaints (*r* = 0.069, *P* = 0.011), mood swings (*r* = 0.066, *P* = 0.014), sexual dysfunction (*r* = 0.074, *P* = 0.006), and urinary symptoms (*r* = 0.076, *P* = 0.005). Modest correlation coefficients nonetheless showed consistent positive associations across symptom domains, indicating weak but statistically significant associations of selected menopausal symptoms.

#### 3.3.3 Multivariable regression analysis

To further evaluate the independent association between adiposity and menopausal symptom severity, multivariable linear regression analyses were performed. As shown in Table 3, higher BMI remained independently associated with greater severity of vasomotor symptoms (β = 0.12, 95% CI 0.03–0.21, *p* = 0.011), sexual dysfunction (β = 0.04, 95% CI 0.01–0.08, *p* = 0.015), and urinary symptoms (β = 0.04, 95% CI 0.01–0.07, *p* = 0.022), after adjustment for WHR. WHR was not independently associated with any symptom domains in the adjusted models (Table 3). Although these associations reached statistical significance, the effect sizes were small, consistent with the weak correlations observed in unadjusted analyses, and are therefore unlikely to be clinically meaningful at the individual level.

**TABLE 3:** Multivariable linear regression analysis of the association between BMI and domain-specific menopausal symptom severity.

Outcome	β (BMI)	95% CI	*p*-value
Vasomotor symptoms	0.12	(0.03–0.21)	0.011
Sexual dysfunction	0.04	(0.01–0.08)	0.015
Urinary symptoms	0.04	(0.01–0.07)	0.022

BMI, body mass index; CI, confidence interval; WHR, waist-to-hip ratio. All values are presented as unstandardized regression coefficients (β) with corresponding 95% confidence intervals (CI). Models were adjusted for waist-to-hip ratio (WHR). *P*-values are two-sided, and values < 0.05 were considered statistically significant.

## 4 Discussion

In this cohort of Chinese women undergoing the menopausal transition, greater symptom severity across several domains was observed in those with higher BMI. We found that the most frequently reported symptoms were hot flashes and night sweats, sexual dysfunction, fatigue, and mood swings. BMI was not associated with symptom occurrence but showed weak associations with greater severity in vasomotor, sexual, and urinary symptoms. WHR, despite being a widely recognized index of central adiposity, showed no significant associations, and chronological age was only weakly related to overall symptom burden, suggesting that factors beyond simple aging or fat distribution may be associated with the menopausal symptom profile (20, 21). In contrast to earlier hypotheses, peripheral aromatization in androgens in adipose tissue has been proposed to protect against vasomotor symptoms (2). These mechanisms may help explain the observed association between higher BMI and greater vasomotor symptom severity. Visceral fat increases insulation and impedes heat dissipation, while producing inflammatory cytokines such as tumor necrosis factor-α and interleukin-6, which act on the hypothalamus to narrow the thermoneutral zone (22). These pro-inflammatory mediators, together with altered leptin and adiponectin signaling, may destabilize the hypothalamic set-point and intensify heat-dissipating responses. Furthermore, obesity is associated with endothelial dysfunction and reduced skin vasodilation, limiting the body’s capacity for efficient heat loss. These mechanisms may explain why women with higher BMI, particularly in early postmenopausal when estrogen levels fluctuate sharply, tended to report more severe vasomotor symptoms despite higher absolute estrogen levels from adipose aromatization (23).

Sexual dysfunction emerged as the second most prevalent symptom, with both prevalence and severity increasing across BMI categories (24). From a pathophysiological standpoint, obesity may impair sexual health through multiple converging pathways. Hyperinsulinemia and dyslipidemia can reduce endothelial nitric oxide production, diminishing genital blood flow and lubrication. Altered sex hormone–binding globulin levels may shift the balance of circulating sex steroids, leading to a mismatch between androgen and estrogen activity. Central adiposity is linked to low-grade inflammation that may affect genital tissue sensation. In addition to physiological effects, psychosocial factors, including body image dissatisfaction, fatigue, and mood disturbance, may further compound sexual dysfunction (25, 26). Evidence from surgical weight loss and intensive lifestyle interventions demonstrates that sexual function scores can improve, suggesting these changes are at least partially reversible and responsive to targeted weight management (14).

Urinary symptoms were likewise more severe among obese women, echoing epidemiological evidence linking elevated BMI to stress and mixed urinary incontinence. The mechanical load of central adiposity increases intra-abdominal pressure, straining the pelvic floor and weakening urethral closure mechanisms. Over time, this pressure, combined with age-related declines in collagen and elastin in pelvic connective tissue, may compromise bladder support. Adipose-driven inflammation may also impair neural control of bladder and urethral function, contributing to urgency and frequency. In our cohort, WHR was not a significant predictor, suggesting that total body mass may have a greater influence on pelvic floor dynamics than fat distribution alone. The observation that urinary symptoms severity, but not prevalence, was associated with BMI implies that weight reduction may lessen symptom intensity even if baseline risk remains. This is consistent with interventional studies showing that modest weight loss can reduce incontinence episodes and improve bladder control, even without full symptom resolution (27). This finding contrasts with earlier studies that associate central adiposity with metabolic risk, suggesting that overall adiposity may play a more significant role in symptom severity in this population. This may partly explain inconsistencies across studies, as the influence of overall versus central adiposity on menopausal symptoms may differ by population and study design. The lack of strong associations between BMI and other menopausal symptoms, such as paresthesia, dizziness, arthralgia, or depressive mood, underscores the selective nature of adiposity’s influence (28). These symptoms may be governed less by metabolic load or mechanical strain and more by neuroendocrine dynamics, neurotransmitter balance, and central nervous system sensitivity to fluctuating estrogen and progesterone (29, 30). Paresthesia and dizziness, for example, may involve transient changes in cerebral blood flow and neuronal excitability, driven by γ-aminobutyric acid and glutamate signaling, independent of adipose-related inflammation. Myalgia may be more closely related to postmenopausal loss of estrogen’s chondroprotective effects, including reduced proteoglycan and type II collagen synthesis within articular cartilage, than to obesity *per se*. Similarly, mood changes are thought to arise from interactions between hypothalamic, pituitary, adrenal axis reactivity, serotonergic and dopaminergic neurotransmission, and psychosocial stressors (31). Obesity can be linked to low-grade inflammation that affects brain function, but the multifactorial nature of mood disturbances suggests they are not solely driven by BMI. This pattern underscores that BMI is not a universal determinant of menopausal health but instead selectively influences symptom clusters tied to thermoregulation, vascular function, and pelvic floor mechanics, where adipose tissue’s endocrine and inflammatory effects are most pronounced (32).

In addition, earlier studies have pointed to strong associations between menopausal symptom severity, metabolic syndrome, and lipid abnormalities (33, 34). The cardiometabolic hypothesis proposes that women with more pronounced vasomotor or urogenital symptoms often present with central obesity, insulin resistance, and elevated blood pressure. The related lipid hypothesis proposes that symptom arises from disturbances in lipid metabolism, such as elevated triglycerides and reduced HDL cholesterol (33, 35). These models indicate that menopausal symptoms may signal underlying metabolic dysfunction. Our results are consistent with this view, as we found that excess adiposity was most strongly related to symptom domains connected to insulin resistance, dyslipidemia, and endothelial dysfunction, which together represent the key features of metabolic syndrome.

From a public health perspective, the observed associations are relevant, as modest differences in symptom severity may influence quality of life in aging populations with increasing obesity (36). Incorporating weight management into perimenopausal care, via dietary advice, structured physical activity, and behavioral support, may be associated with improvements in cardiometabolic health; however, its impact on menopausal symptom severity remains uncertain and requires confirmation in longitudinal or interventional studies (26, 29, 30). Given the stage-specific association between BMI and symptom burden, interventions should be tailored accordingly (37, 38).

This study has several strengths. The sample size was relatively large, menopausal status was clinically verified, and standardized methods were used for anthropometric measurements and symptom assessment, which improves the reliability of the findings. Some limitations should also be noted. Because of the cross-sectional design, causal relationships cannot be established, and longitudinal studies are needed to better understand how adiposity and menopausal symptoms change over time. In addition, all participants were recruited from a single urban center in Eastern China, which may introduce selection bias and limit the representativeness of the study population. Therefore, the findings may not be fully generalizable to women from other regions, rural settings, or different socioeconomic backgrounds. Menopausal symptoms were assessed by self-report, and reporting may be influenced by recall or cultural factors. We also did not collect information on several potentially relevant variables, such as diet, phytoestrogen intake, physical activity, or psychosocial stress. These factors may act as confounders, and their absence limits the ability to determine the independent effect of adiposity on symptom severity. Finally, the absence of hormonal and inflammatory biomarker data limits the ability to explore underlying biological mechanisms. Future studies incorporating longitudinal follow-up and biological measurements may help clarify these relationships. Although several associations reached statistical significance, effect sizes were small, indicating limited clinical relevance. Adiposity measures were associated with menopausal symptoms at the population level. Still, they are unlikely to serve as meaningful predictors of symptom severity in individual women. This distinction highlights the importance of interpreting statistical significance in the context of effect size and clinical relevance.

## 5 Conclusion

Higher overall adiposity was associated with modest increases in the severity of vasomotor, sexual, and urinary symptoms during the menopausal transition. However, given the small effect sizes and cross-sectional design, these findings should be interpreted cautiously and do not establish causal relationships. The clinical relevance of these associations remains uncertain.

## Data Availability

The original contributions presented in the study are included in this article/Supplementary material, further inquiries can be directed to the corresponding author.
