# Supplementary material for: Adiposity and domain-specific menopausal symptom severity in midlife women: a cross-sectional clinical study in eastern china
Source: Front Med (Lausanne). 2026 May 22;13:1831154. doi: 10.3389/fmed.2026.1831154 (PMC13237693; doi:10.3389/fmed.2026.1831154)
Supplement: Supplementary file 1 [file Table_1.docx]

Supplementary Material

**Supplementary Table S1.** Prevalence of peri-menopausal symptoms across BMI-defined study groups

| Symptom | Underweight (n=63) | Normal weight (n=794) | Overweight (n=308) | Obese (n=206) | χ² | P value |
| --- | --- | --- | --- | --- | --- | --- |
| Vasomotor complaints | 50 (79.37) | 590 (74.31) | **246 (79.87)^†^** | **164 (79.61)^†^** | 5.475 | 0.140 |
| Paresthesia | 20 (31.75) | 257 (32.37) | 94 (30.52) | 85 (41.26) | 7.334 | 0.062 |
| Insomnia | 46 (73.02) | 572 (72.04) | 228 (74.03) | 138 (66.99) | 3.174 | 0.366 |
| Mood swings | 40 (63.49) | 507 (63.85) | 215 (69.81) | 147 (71.36) | 6.402 | 0.094 |
| Depressive symptoms | 25 (39.68) | 318 (40.05) | 144 (46.75) | 78 (37.86) | 5.360 | 0.147 |
| Dizziness | 29 (46.03) | 370 (46.60) | 138 (44.81) | 83 (40.29) | 2.668 | 0.460 |
| Fatigue | 47 (74.60) | 570 (71.79) | 222 (72.08) | 148 (71.84) | 0.233 | 0.972 |
| Arthralgia | 30 (47.62) | 461 (58.06) | 185 (60.06) | 109 (52.91) | 5.182 | 0.159 |
| Headache | 21 (33.33) | 297 (37.41) | 112 (36.36) | 79 (38.35) | 0.624 | 0.891 |
| Palpitations | 25 (39.68) | 342 (43.07) | 133 (43.18) | 100 (48.54) | 2.550 | 0.469 |
| Formication | 10 (15.87) | 117 (14.74) | 50 (16.23) | 29 (14.08) | 0.585 | 0.900 |
| Sexual dysfunction | 42 (66.67) | 559 (70.40) | 230 (74.68) | **162 (78.64)^‡^** | 7.480 | 0.059 |
| Urinary symptoms | 14 (22.22) | 252 (31.74) | 101 (32.79) | 77 (37.38) | 5.493 | 0.139 |
| *P < 0.05, *P < 0.001 vs. underweight group; †P < 0.05, ††P < 0.001 vs. normal weight group; ‡P < 0.05, ‡‡P < 0.001 vs. overweight group. BMI groups were defined using WHO Asian cut-offs. Abbreviations: BMI, body mass index; WHR, waist-to-hip ratio | | | | | | |
